# Factors predictive of high-output ileostomy: a retrospective single-center comparative study

**DOI:** 10.1007/s00595-018-1756-2

**Published:** 2018-12-29

**Authors:** Mitsunobu Takeda, Hidekazu Takahashi, Naotsugu Haraguchi, Norikatsu Miyoshi, Taishi Hata, Hirofumi Yamamoto, Chu Matsuda, Tsunekazu Mizushima, Yuichiro Doki, Masaki Mori

**Affiliations:** 1 Department of Gastroenterological Surgery, Osaka University Graduate School of Medicine, 2-2-E2 Yamadaoka, Suita, Osaka 565-0871 Japan; 2 Division of Health Sciences, Department of Molecular Pathology, Osaka University Graduate School of Medicine, 1-7 Yamadaoka, Suita,, Osaka 565-0871 Japan

**Keywords:** High-output stoma, Predictive factors, Ileostomy, Colorectal cancer

## Abstract

**Purpose:**

High-output syndrome (HOS) is a complication of ileostomy, which can affect quality of life significantly; however, its exact cause remains unknown. The aim of this study was to establish the frequency, as well as the preoperative and intraoperative factors predictive of HOS.

**Methods:**

The subjects of this study were 164 consecutive patients who underwent colorectal cancer surgery with ileostomy construction at our institute between January, 2011 and August, 2018. Thirteen patients with postoperative complications reported as causes of HOS, including intraperitoneal abscess, paralytic ileus, and outlet obstruction, were excluded. We used a logistic regression analysis to identify the factors predictive of HOS.

**Results:**

HOS developed in 36 of the 151 patients (23.8%). There were significantly more diabetic patients in the HOS group (*P* = 0.03), but other patient factors such as age, gender, body mass index, and use of daily laxatives were not significantly different between the groups. The HOS group had significantly more cases of total proctocolectomy (*P* = 0.04), but other surgical factors such as operative time, and blood transfusion were not significantly different between the two groups.

**Conclusions:**

These results indicate that diabetes and total proctocolectomy are preoperative predictors of HOS, allowing for the possibility of early intervention via post-surgical treatment.

## Introduction

Temporary ileostomy is being performed frequently in recent years in line with the increase in anal preservation surgery for lower rectal cancer and to prevent suture failure, resulting in additional opportunities for constructing a small bowel stoma [1, 2]. Japan has approximately 150,000 patients with a stoma, including 6000 (4.1%) with an ileostomy [3]. Ileostomy is expected to become even more important to colorectal cancer surgery in the future.

High-output syndrome (HOS) is a complication of small-bowel stoma, which can cause dehydration or renal dysfunction and affect quality of life. The effluent from HOS becomes clinically significant when the daily output exceeds 2000 ml [4], leading to water, sodium, and magnesium depletion, with malnutrition as a late complication. There is limited information about HOS, and most studies [4–8] reporting complications and mortality rates following stoma creation do not include the incidence and causes of HOS. Factors such as intra-abdominal infections, temporary bowel obstruction, residual small bowel < 200 cm, enteritis such as *Clostridium difficile* [9] or salmonella, sudden drug withdrawal (for example, from steroids or opiates), and the administration of prokinetic drugs such as metoclopramide, [8] are thought to contribute to HOS development, but these factors are primarily postoperative, whereas preoperative and intraoperative predictors have not been investigated.

The aim of this study was to establish the frequency, as well as the preoperative and intraoperative factors predictive of HOS. The identification of preoperative predictive factors will enable early intervention and assist with the development of novel therapeutic drugs.

## Methods

All of the experimental protocols described in the present study were approved by the Institutional Ethical Review Committee (UMIN 15046-3: comprehensive agreement) and conform to the provisions of the Declaration of Helsinki.

The subjects of this retrospective case–control study were 164 consecutive patients who underwent elective ileostomy for colorectal cancer between January, 2011 and August, 2018 at Osaka University (Suita, Osaka, Japan). Twenty-nine patients (17.7%) suffered postoperative complications, most of which (75.9%) were Grade 1 or 2 according to the Clavien–Dindo classification [10, 11]. Clavien–Dindo Grade 3 complications were diagnosed in seven patients and included anastomotic leakage (*n* = 2), outlet obstruction [12] (*n* = 4), and ileus (*n* = 1). Among the 164 patients, three had anastomotic leakage, four had intra-abdominal abscess, four had outlet obstruction, and two had ileus. After the exclusion of these 13 patients, because their complications are documented postoperative factors for HOS, 151 patients were the subjects of the final analysis. Adult patients were considered to have HOS if they were referred with a stoma output of more than 2000 ml⁄24 h for three or more consecutive days and biochemical disturbances were imminent [7]. Previous studies defined HOS as an output of ≥ 1500 or 2000 ml; however, there is no consensus on the definition of HOS. Based on reports that HOS with an output of ≥ 2000 ml can cause renal dysfunction [7], this study defined HOS as an output of ≥ 2000 ml to ensure that the criterion is clinically useful. We classified the patients into two groups according to the above definition: HOS (*n =* 36) and non-HOS (*n =* 115). All procedures were performed by highly experienced staff colorectal surgeons.

We compared data on patient demographics, body mass index (BMI), American Society of Anesthesiologists physical status (ASA-PS) classification [13], preoperative chemotherapy, preoperative albumin value, Onodera’s prognostic nutritional index (PNI) [14, 15], tumor location, tumor size, depth of tumor invasion, TNM stage (the eighth edition of Japanese Classification of Colorectal Carcinoma), history of prior surgery, use of a daily proton pump inhibitor (PPI), use of daily laxatives, use of daily steroids, smoking history, high blood pressure, kidney disease, diabetes mellitus, operation time, estimated blood loss, number of lymph nodes harvested, lateral lymph node dissection, total proctocolectomy, perioperative complications, morbidity, C. difficile infection, and the length of hospital stay. Stoma output was measured over a postoperative follow-up period of 6 months for all patients, and any readmissions during this period were recorded.

Ileostomy was created approximately 40–50 cm from away the ileocecal valve for all patients, who had the same postoperative care pathway. Stoma sites were based on a retrospective analysis of operation reports. Early mobilization was encouraged and implemented on postoperative day (POD) 1, as was fluid intake if tolerated. Patients began a light diet on POD 2 and progressed to full meals if tolerated. HOS management is based on the attending doctor’s clinical judgment, and antibiotic or anti-diarrhea agents were given when necessary. Patients were considered fit for discharge when stoma management was mastered and symptoms were stable.

Categorical variables were compared using a *χ*^2^ test. Continuous variables were expressed as medians and ranges and were compared with a Mann–Whitney *U* test or Student’s *t* test. The level of significance was set at *P* < 0.05. A univariate analysis of the categorical data and each individual risk factor for HOS were also performed and the variables considered relevant in the univariate analysis (*P* < 0.05) were entered into a multivariate logistic regression model. The ORs and 95% CIs were calculated for all the variables. All statistical analyses were performed using JMP version 13.0 (SAS Institute Inc., Cary, NC, USA).

## Results

**Fig. 1:**
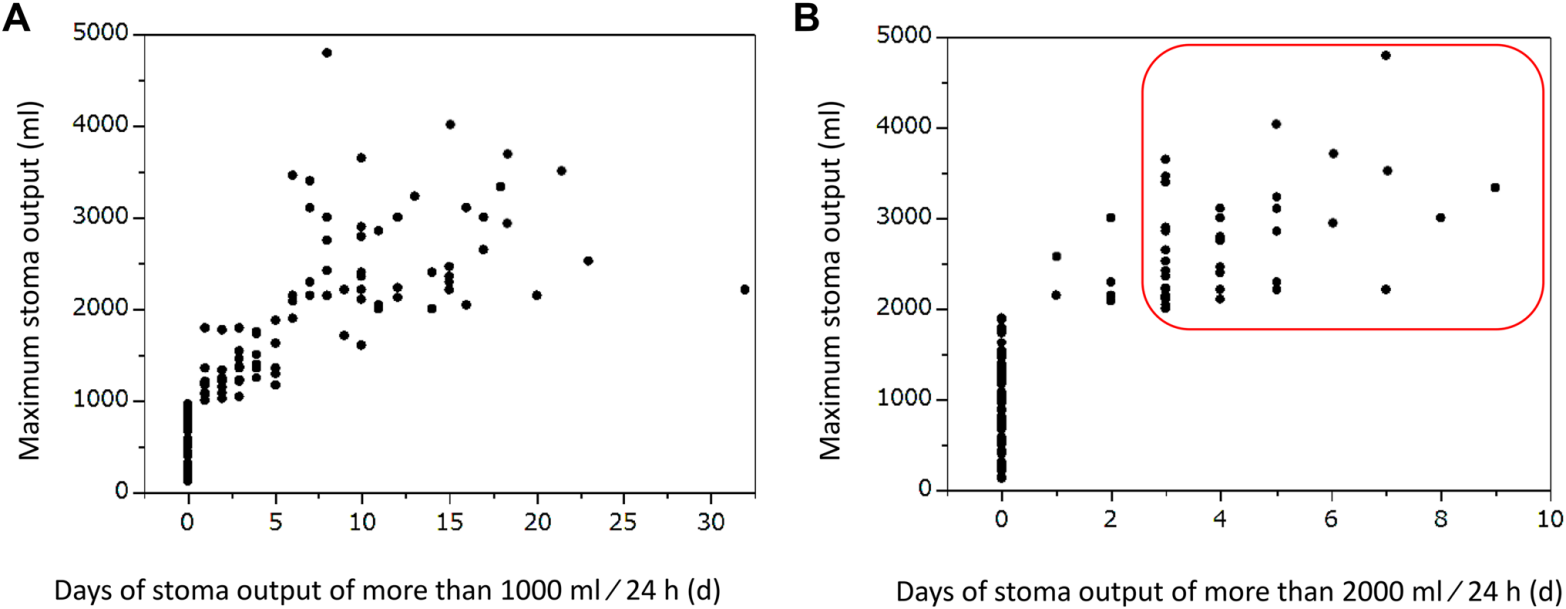
Distribution map of stoma output. The *y*-axis is the maximum stoma output and the *x*-axis is the number of days with output of > 2000 ml/24 h. The framed box indicates the high-output syndrome (HOS) group

HOS developed in 36 of the 151 patients (23.8%). Figure 1 presents the stoma output distribution and days with more than 2000 ml/24 h. The framed box indicates the HOS group. Among the 13 high-risk patients, HOS developed in one of four patients with abdominal abscess, in three of four patients with outlet obstruction, in one of two patients with ileus, and in one of three patients with anastomotic failure. The incidence of HOS among the 13 patients was 46%, which was much higher than the incidence among the 151 patients after excluding the 13 high-risk patients.

The median BMI of the HOS and non-HOS group patients was 22.7 (15.0–38.3) and 23.2 (15.0–32.2) kg/m2, respectively, without a significant difference between the groups. In this study, we did not consider body habitus because we thought that it could be substituted by BMI. The age (59.3 vs 63.0; *P* = 0.11), sex distribution (*P* = 0.97), history of prior surgery (10 vs. 27; *P* = 0.79), and ASA-PS (*P* = 0.82) were also not significantly different between the groups. The patient characteristics did not differ significantly between the groups (Table 1). The patients in this study received only chemotherapy as preoperative treatment because preoperative chemoradiotherapy was not used in our hospital during the study period.

**Table 1:** Characteristics of the patients with ileostomy

Characteristic	Ileostomy		*P* value
	HOS group (*n* = 36)	Non-HOS group (*n* = 115)	
Age (years)	59.0 (26–79)	63.0 (18–84)	0.11
Gender			
Male	21	71	0.97
Female	15	44	
BMI (kg/m2)	22.7 (15–38.3)	22.8 (15.0–32.2)	0.96
ASA status			
1	29	90	0.82
2	5	17	
3	2	8	
Prior surgery	10	27	0.79
Abdominal history	6	17	0.72
Preoperative chemotherapy	7	16	0.80

Data are presented as n or median (range)

*BMI* body mass index, *ASA* American Society of Anesthesiologists

Tables 2 and 3 summarize the patient and surgical factors, respectively. Among the patient factors, preoperative serum albumin (*P* = 0.42), TNM stage (*P* = 0.73), and use of daily laxatives (*P* = 0.21) were not significantly different between the groups (Table 2). Among the surgical factors, operative time (*P* = 0.55), estimated blood loss (*P* = 0.61), and lateral lymph node dissection (*P* = 0.38) were not significantly different between the groups (Table 3). HOS was significantly associated with diabetes mellitus (*P* = 0.03) and total proctocolectomy (*P* = 0.04). HOS was also significantly associated with age (*P* = 0.048), diabetes mellitus (*P* = 0.01), and total proctocolectomy (*P* = 0.03; Table 4) in the univariate analysis, while the multivariate analysis indicated that diabetes mellitus (odds ratio 18.90, 95% confidence interval 3.427–186.1. *P* = 0.002) and total proctocolectomy (odds ratio 15.46, 95% confidence interval 3.173–119.1, *P* = 0.01) were independent significant preoperative factors for HOS (Table 4).

**Table 2:** Patient factors for high-output syndrome (HOS)

Patient factors	Ileostomy		*P* value
	HOS group (*n* = 36)	Non-HOS group (*n* = 115)	
Preoperative albumin value (g/dl)	3.9 (1.9–4.6)	4.0 (2.8–5.1)	0.42
Onodera’s PNI	47.0 (22.8–55.4)	46.5 (30.2–64.0)	0.48
Depth of tumor invasion			
T0–2	22	71	0.84
T3–4	14	44	
TNM stage			
0–II	25	76	0.73
III–IV	11	39	
Use of daily PPI	5	9	0.78
Use of daily laxatives	3	5	0.21
Use of daily steroid	1	3	0.89
Smoking history	8	27	0.64
Heart disease	2	8	0.73
High blood pressure	7	19	0.55
Kidney disease	3	6	0.61
Diabetes mellitus	10	2	0.03

Data are presented as n or median (range)

*PNI* Prognostic Nutritional Index, *PPI* proton pump inhibitor

**Table 3:** Surgical factors associated with high-output syndrome (HOS)

Surgical factors and complications	Ileostomy		*P* value
	HOS group (*n* = 36)	Non-HOS group (*n* = 115)	
Operative time (min)	429 (149–961)	425 (180–917)	0.55
Estimated blood loss (ml)	150 (0–1850)	120 (0–5660)	0.61
Blood transfusion	2	6	0.29
Lateral lymph node dissection	9	22	0.38
Conversion of laparotomy	1	2	0.82
Total proctocolectomy	6	1	0.04
Clostridium difficile infection	0	0	–

Data are presented as n or median (range)

**Table 4:** Univariate and multivariate analyses of the preoperative predictors of high-output syndrome (HOS)

	Univariate analysis			Multivariate analysis		
	OR	95% CI	*P* value	OR	95% CI	*P* value
Age	5.889	1.411–19.20	0.048	2.033	0.311–13.81	0.47
Gender	0.889	0.414–1.931	0.77			
BMI	2.096	0.992–5.278	0.10			
Onodera’s PNI	1.051	0.978–3.275	0.13			
Diabetes mellitus	11.22	2.341–53.74	0.01	18.90	3.427–186.1	0.002
Operative time	0.821	0.398–1.779	0.84			
Total proctocolectomy	10.07	3.473–47.26	0.03	15.46	3.173–119.1	0.01

*OR* odds ratio, *CI* confidence interval

In the multivariate analysis of all 164 patients, including the 13 with HOS-related disease, outlet obstruction (odds ratio 4.452, 95% confidence interval 1.101–29.68, *P* = 0.041), diabetes mellitus (odds ratio 5.735, 95% confidence interval 1.112–105.1, *P* = 0.034), and total proctocolectomy (odds ratio 15.46, 95% confidence interval 1.024–25.13, *P* = 0.048) were independent significant factors for HOS (Table 5). Even when all 164 patients were analyzed, HOS was significantly associated with diabetes mellitus and total proctocolectomy.

**Table 5:** Univariate and multivariate analyses of the preoperative predictors of high-output syndrome (*n* = 164)

	Univariate analysis			Multivariate analysis		
	OR	95% CI	*P* value	OR	95% CI	*P* value
Age	0.921	0.332–2.748	0.877			
Intra-abdominal abscess	3.095	0.851–18.83	0.092			
Outlet obstruction	5.274	1.179–12.56	0.029	4.452	1.101–29.68	0.041
Ileus	3.851	0.978–14.95	0.081			
Anastomotic leakage	1.627	0.450–2.222	0.424			
Diabetes mellitus	7.637	1.534–138.2	0.008	5.735	1.112–105.1	0.034
Total proctocolectomy	4.811	1.611–31.28	0.042	3.946	1.024–23.13	0.048

*OR* odds ratio, *CI* confidence interval

Patients were discharged from hospital after a mean period of 40.7 ± 12.5 and 21.2 ± 7.8 PODs (mean ± SD) in the HOS group and non-HOS groups, respectively. The hospitalization period was significantly longer in the HOS group (*P* = 0.0002).

## Discussion

There are few reports of HOS in the literature, and most have focused on drainage management after surgery or hospital discharge [4–8]. Some studies [4–9] suggest that HOS is caused by conditions such as intra-abdominal abscess and intestinal obstruction, but this is most likely because these conditions coincide with postoperative complications. In other words, it is possible that HOS development occurs secondary to intra-abdominal abscess and other such conditions because of the resulting paralytic ileus that prevents absorption of small intestinal drainage. Therefore, no study has identified the specific cause of HOS itself. Yet, an identified cause could lead to early therapeutic intervention to prevent HOS and eliminate the need for long-term hospitalization and management, reducing medical expenses. This requires identification of the preoperative factors that predict HOS onset.

We identified, retrospectively, the preoperative factors predictive of HOS onset in patients without the postoperative complications previously reported to cause HOS, such as intra-abdominal abscess, paralytic ileus, outlet obstruction, or suture rupture [4–8]. To our knowledge, this is the first study to identify potential preoperative predictors of HOS; specifically, diabetes and total colectomy.

HOS developed in 23.8% of the patients in this study, which is slightly higher than the 16% reported in previous studies [4, 8]. This may be because previous studies included both ileostomy and colostomy, or potential differences in HOS definitions [4, 8]. The mechanism by which total colectomy causes HOS probably involves various overlapping factors, but one of these could be bile acid deficiency. Total proctocolectomy prevents the reabsorption of bile acids absorbed by the ileocecum, and the resulting decrease in bile acid pools inhibits lipid absorption [16, 17]. Consequently, unabsorbed long-chain fatty acids are hydroxylated or desaturated by anaerobic intestinal bacteria, triggering the secretion of fluid and electrolytes [17], which may lead to the development of HOS. Bile acid deficiency may also cause changes in the intestinal flora that increase intestinal drainage [17]. Diabetes could potentially lead to HOS through the following mechanism: autonomic nervous system impairment caused by diabetes decreases motor function [18], which causes abnormal proliferation of intestinal bacteria primarily in the upper small intestine, producing gas in the intestine and increasing intestinal pressure [18, 19]. The physical stimulus of this pressure may cause HOS.

Assuming that HOS develops through the above mechanisms, it may be possible to alleviate HOS in patients who have undergone total proctocolectomy by supplementing their bile acids (ursodeoxycholic acid). However, this requires a clinical trial to establish bile acid dose adjustments before and after surgery. Diabetes is associated with all postoperative complications, but strict preoperative and operative management of blood glucose levels may also reduce the incidence of HOS.

None of the patients in this study needed readmission, but Baker et al. [8] reported a 5% readmission rate for HOS, and Nightingale et al. [4] reported a rate of 50%. This may be because patients in Japan are typically hospitalized for longer than those in other countries [1, 20, 21], and HOS just prolonged their hospitalization. Measures such as those proposed above could reduce the incidence of HOS, prevent prolonged hospitalization, and improve quality of life.

The limitations of this study were the fact that it was conducted in a single center and the sample size was small, which means that the conclusions cannot be considered definitive. Moreover, the volumes of water intake and infusion were not taken into account.

In conclusion, we identified diabetes and total proctocolectomy as preoperative predictors of HOS. Establishing the preoperative predictive factors will enable early intervention by post-surgical treatment and may assist with the development of novel therapeutic drugs.
